# Approaches in studying the pharmacology of Chinese Medicine formulas: bottom-up, top-down—and meeting in the middle

**DOI:** 10.1186/s13020-018-0170-4

**Published:** 2018-03-21

**Authors:** Tao Huang, Linda L. D. Zhong, Chen-Yuan Lin, Ling Zhao, Zi-Wan Ning, Dong-Dong Hu, Man Zhang, Ke Tian, Chung-Wah Cheng, Zhao-Xiang Bian, for MZRW Research Group

**Affiliations:** 1 Institute of Brain and Gut Research, School of Chinese Medicine, Hong Kong Baptist University, Room 307, Jockey Club School of Chinese Medicine, 7 Baptist University Road, Kowloon, Hong Kong, Hong Kong SAR China; 2 Hong Kong Chinese Medicine Clinical Study Centre, Hong Kong Baptist University, Room 307, Jockey Club School of Chinese Medicine, 7 Baptist University Road, Kowloon, Hong Kong, Hong Kong SAR China; 3 YMU-HKBU Joint Laboratory of Traditional Natural Medicine, Yunnan Minzu University, Kunming, 650500 China; 4 Guangzhou Research Institute of Snake Venom, Guangzhou Medical University, Guangzhou, 510000 China

**Keywords:** Bottom-up, Chinese medicine formula, Focused network pharmacology, Pharmacometabolomics, Top-down

## Abstract

Investigating the pharmacology is key to the modernization of Chinese Medicine (CM) formulas. However, identifying which are the active compound(s) of CM formulas, which biological entities they target, and through which signaling pathway(s) they act to modify disease symptoms, are still difficult tasks for researchers, even when equipped with an arsenal of advanced modern technologies. Multiple approaches, including network pharmacology, pharmaco-genomics, -proteomics, and -metabolomics, have been developed to study the pharmacology of CM formulas. They fall into two general categories in terms of how they tackle a problem: bottom-up and top-down. In this article, we compared these two different approaches in several dimensions by using the case of MaZiRenWan (MZRW, also known as Hemp Seed Pill), a CM herbal formula for functional constipation. Multiple hypotheses are easy to be proposed in the bottom-up approach (e.g. network pharmacology); but these hypotheses are usually false positives and hard to be tested. In contrast, it is hard to suggest hypotheses in the top-down approach (e.g. pharmacometabolomics); however, once a hypothesis is proposed, it is much easier to be tested. Merging of these two approaches could results in a powerful approach, which could be the new paradigm for the pharmacological study of CM formulas.

## Background

**Fig. 1:**
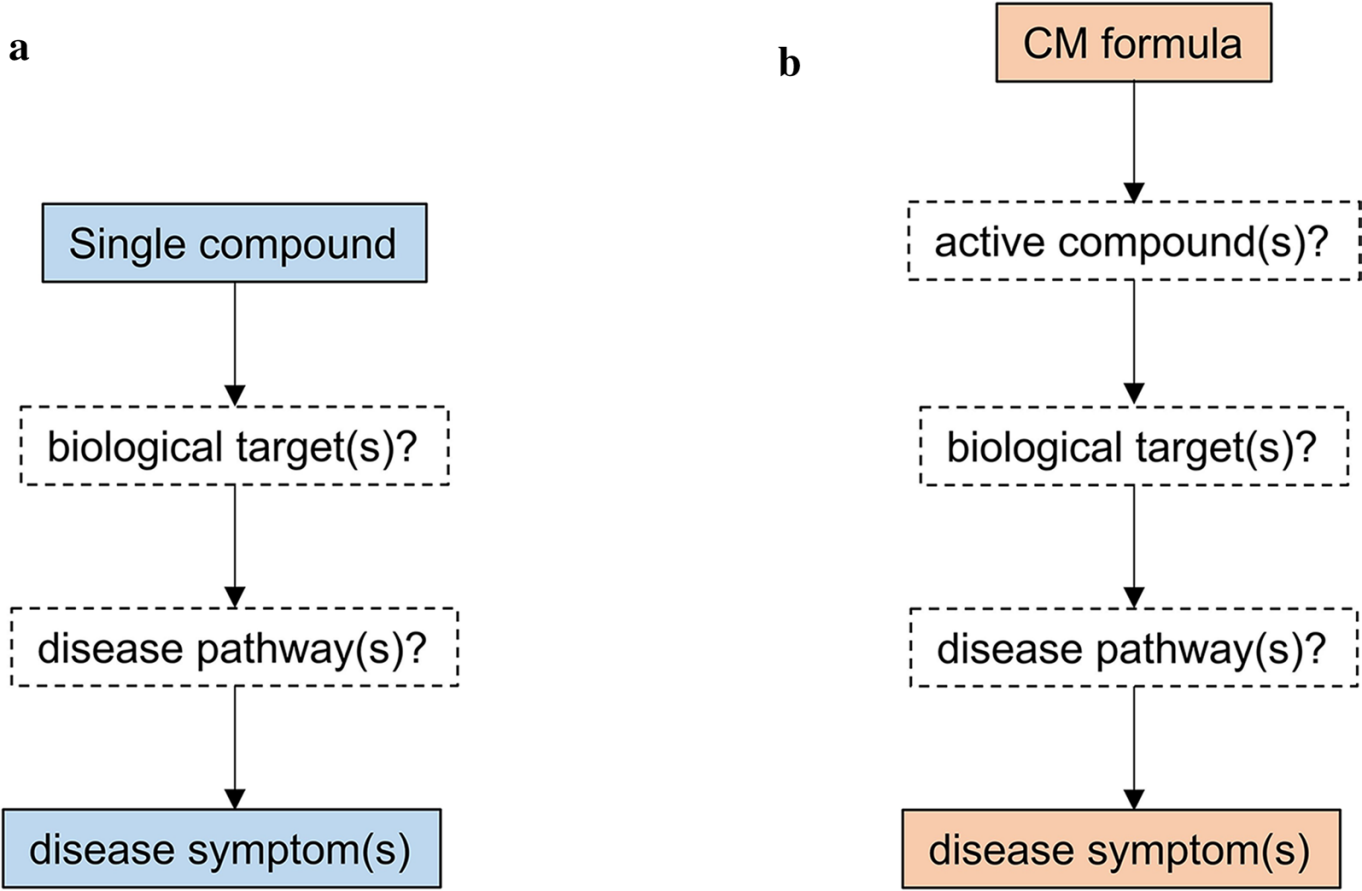
The comparison of pharmacology study content between single compound and CM formula. In investigating the pharmacological actions of single compound (**a**), the researchers are set out to figure out which biological target(s) and which disease pathway(s) will be affected by the compound. While for CM formula (**b**), besides the biological target(s) and disease pathway(s), the active compound(s) which are responsible for disease modification, are also required to be identified. In some cases, the combinational effect of these active compounds are also needed to be elucidated

Unknown active constituents and unclear mechanism-of-actions have sparked criticism when Chinese medicine (CM) formula is getting more popular today [1, 2]. Thus, investigating the pharmacology is important to the modernization of CM formula. However, the pharmacological study of a CM formula is much more complicated than that of a single compound. With a single compound study, researchers need only determine which biological target(s) it acts on, and which disease pathway(s) it alters (Fig. 1a). With a formula study, there is much more to be done and many more factors to be considered. Firstly, the CM formula is comprised of several herbs, each of which contains hundreds, possibly thousands, of compounds, many of which could be unique to that herb. Secondly, not all the compounds from herb are involved in the pharmacological activity—some of them are removed during preparation, while some of them are just passed by. Thirdly, most compounds from herbs are weak modulators of biological targets, thus the effect of an individual compound is hard to determine. Fourthly, the herbal compounds may have multiple pharmacological actions, some of which are not directly correlated with symptom improvement; to identify the targets and pathways that are truly involved is not easy. Lastly, the complex interactions (synergistic or antagonistic) between herb compounds are hard to determine.

**Fig. 2:**
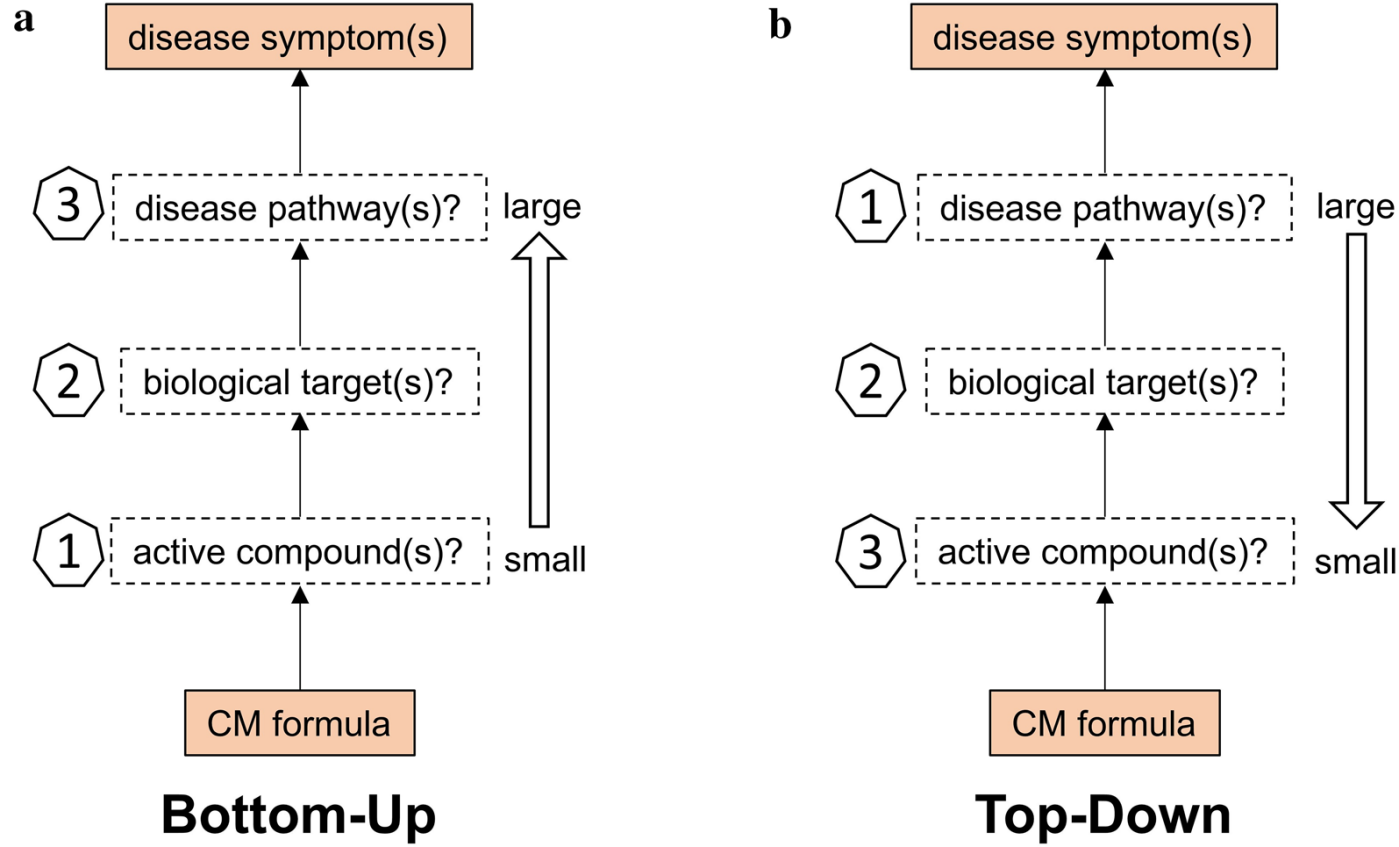
The comparison of two different approaches in studying the pharmacology of CM formula: bottom-up and top-down. In the bottom-up approach (**a**), the pharmacology of CM formula is investigated from small scale (compound) to large scale (pathway). The CM formula is firstly breakdown into hundreds or thousands of compound with various experimental or computational methods. Then the biological target(s) of these compounds are identified via literature search, in silico inference, and/or experimental validation. Finally, the affected disease pathway(s) were studied. In contrast, in the top-down approach (**b**), the pharmacology of CM formula is investigated from large scale (pathway) to small scale (compound). The CM formula is treated as a whole and the affected disease pathway(s) are elucidated firstly. Then the biological target(s) are proposed and an assay method is established based on this target(s). Finally, the active compound(s), which are responsible for acting on this target(s) and altering the disease pathway(s), are screened and identified with the established assay. (The arrow of **b** should be changed?)

Multiple approaches have been utilized and developed for investigating the pharmacology of CM formula, including network pharmacology, pharmaco-genomics, -proteomics, and -metabolomics. These approaches have been successfully applied in studying the pharmacology of the Liu-Wei-Di-Huang pill, Qing-Luo-Yin, and other CM formulas [3–11]. In particular, there are reviews discussing the theory, methodology and applications of CM network pharmacology [12–19]. We used several of these methodologies to investigate the pharmacology of a CM formula MaZiRenWan (MZRW, also known as Hemp Seed Pill) [20, 21]. Based on the nature, we observe that most of these approaches fall into one of two categories in terms of how they tackle the problem: bottom-up, or top-down (Fig. 2).

In the context of medicinal herb research, by bottom-up, we mean starting with the many smaller units, i.e. isolated constituents, and determining their larger role in a disease pathway (Fig. 2a). By top-down, we mean starting with the disease pathway, and determining which constituents are involved in regulating it (Fig. 2b). These two contrasting approaches are equally effective—and are seen in other contexts such as nanotechnology, neuroscience, psychology, public health, ecology, management, and organization [22]. For example, in cognitive process, bottom-up cognition is focusing on details primarily, then the whole landscape. While a top-down approach is used by the person who focus on the big picture first and from that figure out details to support it [23]. In this article, we will compare these two distinct approaches in the investigation of pharmacology of one CM formula, MZRW for functional constipation (FC).

MZRW is an herbal formula for constipation from traditional chinese medicine (TCM). About 2000 years ago, MZRW was firstly recorded in *Discussion of Cold*-*Induced Disorders* (*Shang Han Lun*) [24, 25]. It is comprised of six herbs, namely *Fructus cannabis* (*Huo Ma Ren*), *Radix et rhizoma rhei* (*Da Huang*), *Semen Armeniacae Amarum* (*Ku Xing Ren*), *Radix paeoniae Albo* (*Bai Shao*), *Cortex magnolia officinalis* (*Hou Pu*), and *Fructus aurantii immaturus* (*Zhi Shi*) [26]. In TCM theory, MZRW can drain heat, unblock the bowel, promote the movement of Qi, and moisten the intestines [26].

We chose MZRW because a systematic review of the published literature showed that MZRW is the most frequently used TCM formula for constipation [27] yet there is little if any strict clinical evidence of its efficacy. To that end, we demonstrated that MZRW is significantly better than placebo in improvement of bowel movement during drug treatment, while such effect is more sustainable than placebo during 8 weeks follow-up, in the randomized, placebo-controlled clinical study with 120 FC patients [26]. Recently, we have finished a larger clinical study including 291 FC patients to compare the efficacy of MZRW with that of Senna (commonly used laxative in Hong Kong) and placebo [28]. The results showed that, both MZRW and Senna are better than placebo during the treatment period; while the efficacy of MZRW is more sustainable than that of Senna and placebo in the follow-up period. We also identified ten major compounds from MZRW in rat plasma by UPLC–MS/MS [29] to facilitate the pharmacokinetic study of MZRW in healthy volunteers [30].

On top of this solid clinical evidence and pharmacokinetic data, we set out to elucidate the pharmacology of MZRW for FC. We tried different methodologies to determine (1) which active compound(s) are in MZRW, and how they act (2) on which biological target(s), (3) through which signaling pathway(s) to alter the bowel movement, as slow bowel movement is the major symptom of FC patients. Doing this work eventually we realized that every methodology has its own advantages and disadvantages, but they can be compared in an efficient way: bottom-up versus top-down (Table 1). In the following sections we will first describe these two different approaches; then describe their application in the analysis of MZRW; and conclude with the take-home lessons for doing similar research on other CM formulas.

**Table 1:** Bottom-up and top-down approaches in pharmacological research of CM formula

Approach	Bottom-up	Top-down
Representative methodology	Network pharmacology	Pharmacogenomics, Pharmacoproteomics, Pharmcometabolomics
Question solving order	From small (compounds) to large (disease pathways)	From large (biological pathways) to small (compound)
Hypothesis forming	Easy	Hard
Multiple hypotheses producing	Yes	No, usually single
Hypothesis testing	Hard	Easy

## Bottom-up approaches

In the bottom-up approach, researchers start with compounds, look for biological targets, and work toward understanding the biochemistry of the disease pathway(s) (Fig. 2a).

Network pharmacology is the representative methodology of the bottom-up approach. Firstly, the compounds have been identified as constituents of these herbs of CM formula via literature/database search, and/or LC–MS identification, etc. Secondly, the known biological targets of these compounds are collected by literature/database search and/or predicted by various computational tools, such as inverse docking, bioactivity spectra analysis, and chemical similarity searching. Thirdly, the biological targets are used to build a network based on a molecular interaction database, and the relevant signaling pathways can be focused on with enrichment analysis tools. Finally, by using this network, the hypotheses, that which compound(s) could modify the disease symptoms through which target(s)/pathway(s), are generated. Then each potentially active compound is tested to determine whether it, in fact, affects the pathways involved in the disease. In general, it is easy to generate multiple hypotheses with network pharmacology. However, inevitably, a number of these hypotheses are just false positives, and testing so many hypotheses is mission impossible (Table 1), as can be seen with our work on MZRW [21].

**Fig. 3:**
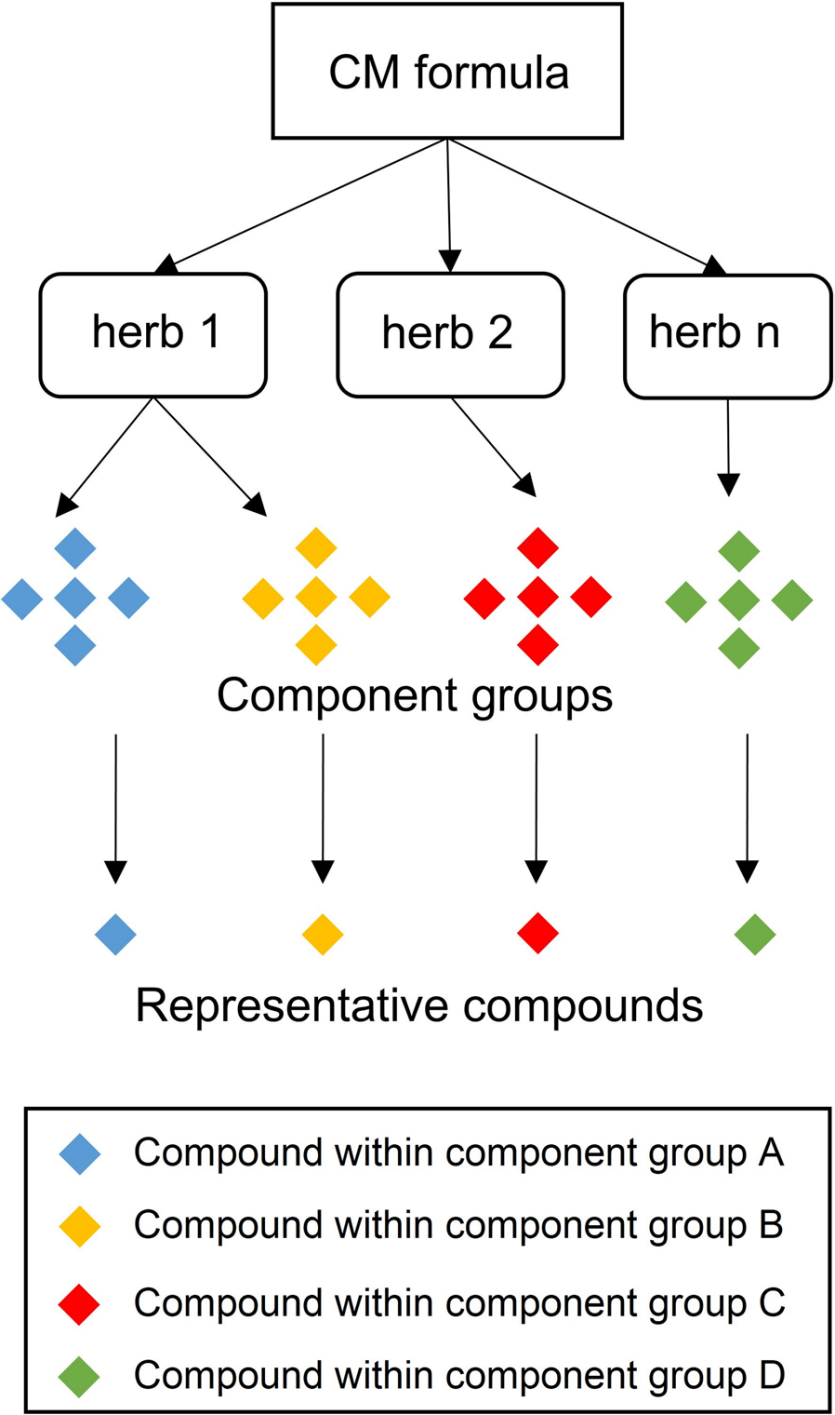
The “representative compound” concept to reduce the redundancy of active compounds in network pharmacology. The CM formula could be breakdown in a hierarchy manner, first into herbs, then the component groups (A, B, C, D, etc.), finally into the representative compounds. In one of component group, compound are similar to each other in chemical structure, and most of them are from a single herb. Considering that structurally similar compounds usually have similar bioactivity, a representative compound of this component group can be used to study the pharmacological action of that component group

The first problem is the huge number of compounds in any herbal formula. There are only six herbs in MZRW; however, the number of unique compounds in these six herbs, based on a database constructed from a literature search, is greater than one thousand. Due to one compound could act on multiple targets, one thousand compounds would result in ten thousand hypotheses; it would be impractical if not impossible to test them all. Thus, we used several ways to reduce the number of candidate compounds. Firstly, the compounds that were detected in extracts and biological samples with LC-MS were kept, while the remaining were discarded. This method resulted in 97 candidate compounds, a feasible number for testing. Secondly, to reduce redundancy, we used chemical structure clustering analysis to classify the 97 compounds into small component groups. Within each component group, the candidate compounds are similar to each other. Based on the well-known observation that “similar compounds have similar bioactivities” [31], a compound was selected from each component group and its pharmacological action was considered representative of that group (Fig. 3). Thirdly, we used rat colonic segments in an organ bath to determine which, if any, of these representative compounds enhanced colonic motility, in the phenotypic symptom we had chosen to model FC. Finally, we had 5 representative compounds that were active in reducing FC: emodin, amygdalin, albiflorin, honokiol, and naringin.

The second problem with network pharmacology in particular and the bottom-up approach in general is similar to the first: there are a huge number of hitting biological targets. Within a literature/database search and chemical similarity search [32], we found 10 + targets for each of the 5 representative compounds. Although there might be some novel targets related to disease modification, we still thought that the number of biological targets that could explain the pharmacological actions of MZRW was overestimated. To solve this problem, we checked the target-disease link with a literature search. Finally, 7 targets (ACHE, ESR2, CYP19A1, PTGS1, PTGS2, ADORA1, CNR1), either referenced in the literature or predicted by computational tool, were found have direct link with constipation.

The third problem is the large number of predicted pathways. Previous reported network pharmacology studies suggest huge networks involving dozens or hundreds signaling pathways. However, most of these pathways are not directly related with disease modifications, and testing such pathways would cause time and funding waste in experimental validation. For our purposes, only the disease pathways matters. With all the efforts described above, we were able to minimize the number of predicted pathways into five disease pathways: acetylcholine-, estrogen-, prostaglandin-, cannabinoid-, and purine. All of them have been shown to be related with bowel movement evidenced by human and animal studies.

In summary, in the traditional network pharmacology, a huge number of compounds, targets, and pathways generates too many hypotheses to be tested in real time. With MZRW, only by selecting representative compounds, targets and pathways were we able to generate a feasible number of hypotheses for testing. This new approach was named after “focused network pharmacology” [21].

## Top-down approaches

In the top-down approach, the researchers solve the key questions in the large-to-small manner: from disease pathway(s), to biological target(s), to compound(s) (Fig. 2b). Compared with the bottom-up approach (network pharmacology), the top-down approach is relatively less used in studying the pharmacology of CM formulas [33, 34].

Representative methodologies of top-down approach are pharmaco-omics, including pharmacogenomics, pharmacoproteomics, and pharmacometabolomics. Pharmaco-omics has two meaning. The first would be to study the effects of a CM formula on specific biomarkers (genes, proteins, metabolites, etc.) during drug treatment. The second would be study of the effects of a specific genotype (or protein/metabolite level) on the efficacy of treatment CM formula. Here we use the first definition. Firstly, change of biomarker levels in samples (biofluids or tissues) before and after drug treatment are measured with genomics, proteomics or metabolomics technologies. Significant altered biomarkers are attributed to the drug treatment effect. To select specific biomarkers for further study, the biomarker alteration profile of the drug treatment group is compared with that of placebo group, or positive drug group. The effect of a CM formula on such specific biomarkers and associated disease pathway is validated through animal study. Secondly, within the focused disease pathway, one protein is proposed as a candidate target on which the CM formula acts. Thirdly, by using this target, an easy-to-handle screening assay is established and used to identify active compounds from the CM formula. Although it is quite hard, after a few hypotheses are suggested, they are readily validated through animal study (Table 1). We will explain the process in detail with our pharmacometabolomic (top-down) study of MZRW [20].

In the first step, we used samples and data from our previous clinical study comparing the efficacy of MZRW with that of Senna and placebo in 291 FC patients [28]. During this study, we randomly collected serum samples before and after treatment. The serum samples were subjected to untargeted metabolomics analysis, and about 2700 fragments were found in positive and negative modes. The degree of change in these fragments before and after treatment in each patient was calculated, and these alterations were correlated with the improvement of complete spontaneous bowel movement (CSBM), the major endpoint of this clinical study. By comparing the correlation profile in three groups, we found several fragments were significantly correlated with the CSBM improvement in MZRW group, but not in Senna or placebo groups. After analysis with Metabolite and Tandem MS Database (https://metlin.scripps.edu), 15 of these fragments were identified, and 4 of them were found to be structurally closely related to the fatty acid amide (FAA). The one with the most significant correlation of MZRW efficacy was oleamide, an endogenous FAA which is well-known for intestinal motility regulation [35]. Based on this complex analysis, we were able to link MZRW with the oleamide signaling pathway (the disease pathway).

In the second step, we tested which proteins in the oleamide signaling pathways are affected by MZRW. In the mouse models, we found that, the colonic fatty acid amide hydrolase (FAAH) was significantly up-regulated in colon tissue after MZRW treatment. Thus, we identified FAAH as the major target of MZRW for FC.

To complete the third step, we are establishing a cell-based assay to test which compounds from MZRW may regulate FAAH to control the level of oleamide in the colon. At this rate, we predict it will take several years to finish the compound screening; however, we have confidence we will ultimately succeed.

In summary, the most difficult part of the top-down approach is identifying which disease pathway is affected by the CM formula. Sample collection can take years and the data analysis is complex; however, once the hypothesis is generated, it is easy to be tested. We believe that advances in technology/computation will speed things up and make the top-down approaches are more feasible.

## Conclusions

In this article, we compared the bottom-up and top-down approaches in the study of CM herbal formula, particular with the example of MZRW for FC. The bottom-up approach starts with compounds and ends with biological pathways or networks; while the top-down approach begins with pathways and ends with individual compounds. Multiple hypotheses are readily proposed in the bottom-up approach (e.g. network pharmacology); but these hypotheses are hard to test due to the huge numbers of compounds/targets/pathways and high false positive predictions. In contrast, long-term sample collection and complex data analysis makes it hard to suggest hypotheses in the top-down approach; however, once a hypothesis is found, it is much easier to be tested. In the past decade, the bottom-up approach has been frequently applied to CM formulas, but the impact was restricted because it is relatively less testable. In the future, the top-down approach would be more favorably adopted by the researchers, because it is much more testable and will deliver more accurate and concentrated results.

We also image a hybrid model where the bottom-up and top-down approaches meet in the middle. This new approach, utilizing the predicted and validated compound-target link in the bottom-up approach, in the compound screening process of the top-down approach, could reduce the time and cost of identifying the active compounds. The merging of two distinct approaches, bottom-up and top-down, will generate a powerful new approach in the study of the pharmacology of CM formula in the near future.

## Abbreviations

CM: Chinese medicine
CSBM: complete spontaneous bowel movement
FAA: fatty acid amide
FAAH: fatty acid amide hydrolase
FC: functional constipation
MZRW: MaZiRenWan
TCM: traditional Chinese medicine
